# Is the fast-track process efficient and safe for older adults admitted to the emergency department?

**DOI:** 10.1186/s12877-020-01536-5

**Published:** 2020-04-28

**Authors:** B. Gasperini, F. Pierri, E. Espinosa, A. Fazi, G. Maracchini, A. Cherubini

**Affiliations:** 1 Department of Geriatrics and Rehabilitation, Santa Croce Hospital, Azienda Ospedaliera Ospedali Riuniti Marche Nord, Viale Vittorio Veneto 2, 61032 Fano, Italy; 2 Department of Economics, Statistical Section, University of Perugia, Perugia, Italy; 3 Agenzia Regionale Sanitaria-Regione Marche, Senigallia, Ancona Italy; 4 Emergency Department Ospedale Principe di Piemonte, Area Vasta 2, Senigallia, AN Italy; 5 Geriatria, Accettazione geriatrica e Centro di ricerca per l’invecchiamento, IRCCS INRCA, Ancona, Italy

**Keywords:** Emergency department, Fast-track, Older adults, Length of stay

## Abstract

**Background:**

The efficiency of the fast-track (FT) process in the management of patients in Emergency Departments is well demonstrated, but there is a lack of research focused on older adults. The aim of our study was to verify whether the FT process is efficient and safe for older adults admitted to ED.

**Methods:**

Observational case-control single-centre study.

**Results:**

Five hundred four cases and 504 controls were analysed**.** The mean age was 75 years, and there was a predominance of women. In total 96% of subjects were classified with a “less-urgent” tag. The length of stay was significantly lower in the fast-track group than in the control group (median 178 min, interquartile range 184 min, and 115 min, interquartile range 69 min, respectively, *p* < 0.001), as well as the time spent between the ED physician’s visit and patient discharge (median 78 min, interquartile range 120 min, and median 3 min, interquartile range 6 min, respectively, *p* < 0.001). There weren’t any increases in the number of unplanned readmissions within 48 h, 7 days and 30 days.

**Conclusions:**

The fast-track appears to be an efficient and safe strategy to improve the management of older adults admitted to the ED with minor complaints.

## Background

Fast-track (FT) is a process developed to manage patients admitted to Emergency Departments (ED) with non-urgent complaints more effectively [1]. It consists of a separate pathway for patients with less serious conditions who can be treated and discharged more quickly.

Patients suitable to be managed via the fast-track process are identified by the triage nurse using specific inclusion criteria based on presenting problems and the triage category. The literature shows that the advantages of this approach are a shorter waiting time, a shorter ED length of stay (LOS), a decreased rate of patients leaving the ED without being seen and decreased ED overcrowding [2], without increasing unplanned readmissions or mortality [3] as well as affecting waiting times and ED LOS for other ED patients [4].

The ED LOS should be as short as possible for older adults. While ED LOS should be kept between four and eight hours for the general population [5], it has been demonstrated that after 6 hrs the risk of older adults developing adverse outcomes is significantly increased [6, 7]. Indeed, the ED LOS is associated with a higher risk of delirium [8, 9], which is often unrecognised [10], an increased rate of in-hospital admissions and in-hospital LOS, and an increased mortality rate, also after discharge [11]. Despite this evidence, older adults often have longer waiting times [12].

It is therefore a priority to develop safe strategies to reduce the ED LOS of older adults. Among the possibilities, there is the implementation of the fast-track process.

To our knowledge, there is a lack of research focused on the ED management of older adults using the FT process.

The first goal of our study was to verify whether the FT process is effective to reduce the ED LOS of older adults. The second goal was to assess its safety, considering the rate of early unplanned readmissions after discharge from the ED..

## Methods

### Study design and setting

This is an observational case-control single-centre study.

Data were extracted from the administrative database of ED visits at the 200-bed, Principe di Piemonte Hospital in Senigallia (AN), Italy. This ED had an annual census of about 30,000 patients per year.

The FT process was implemented in 2011. For this reason, we compared subjects admitted between January 1 and December 31, 2010, with those admitted between January 1 and December 31, 2012, i.e. the year before and the year after the implementation of the FT process.

The research was conducted in accordance with ethical standards. The Ethics Committee of Marches Region does not require formal approval for observational studies that do not involve the use of drugs.

### Selection of participants

**Fig. 1:**
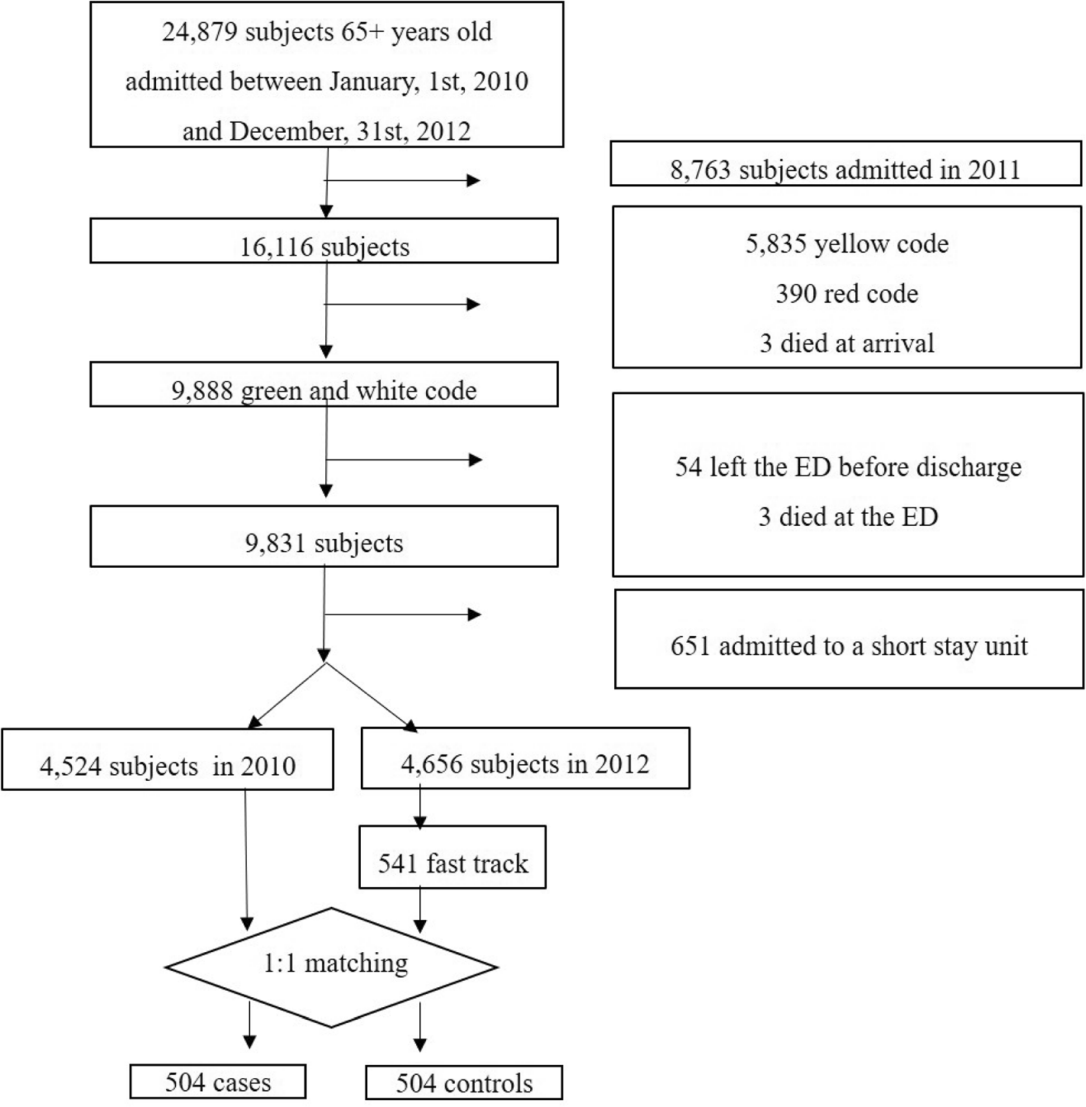
Flow chart of sample selection. Cases were selected among fast-track process patients admitted in 2010. Controls were selected among white and green tag patients admitted in 2012

The sample selection is shown in Fig. 1. Subjects aged 65 years, or more, were considered.

For patients admitted in 2010, those with a high priority level at admission (red and yellow in the Italian coding system), or who left the ED before discharge or died at arrival were excluded. Moreover, patients admitted to the Short Stay Unit were also left out, because in the administrative database the time spent in this unit is included in the ED LOS. Patients admitted in a Short Stay unit are those who need an observation period before a decision can be made concerning their final destination, i.e. admission or discharge to be discharged (i.e. patients with chest pain or those who experienced after a head injury). Short stay unit also admit patients who need an intensive care observation before to being admitted in a ward or, in some instances, patients who should be admitted to the hospital, but the bed is not immediately available.

Data were arranged according to a matched case-control 1:1 design.

All patients triaged at fast-track during the year 2012 (541) represent the cases used, while controls were patients admitted to ED with non-urgent complaints (a white or green colour tag at admission) from January 1 to December 31, 2010, before the fast-track process implementation (*n* = 4524). Data stratification, according to sex, age, triage category and ICD-9 discharge diagnosis gives 175 strata for cases and 819 for controls. The final matched 1:1 data set count was 1008 observation (504 observation for cases and 504 observation for controls) and 141 strata.

### Fast-track process

The fast-track process is applicable when the patient is admitted to the ED with less urgent or non-urgent priority (green or white tags) for a specific complaint that needs a specialist consultation (gynaecologist, ophthalmologist, ear-nose-throat specialist, dental practitioner) or in cases of minor trauma (involving fingers, feet, ankles, elbows or hands). In these cases, after the triage evaluation, colour code assignment and classification of the reason for the ED visit, the triage-skilled nurse can refer the patient to a specialist or request X-ray examination. There is a specific checklist to verify the appropriateness of the X-ray examination. The patient can refuse the fast-track process; therefore, written informed consent is required. An English version of the consent form is available for non-Italian patients.

After the specialist visit or when the X-ray report is available, the patient is evaluated by the ED physician, who can request more exams or consultations, discharge the patient or admit the patient to a ward. At discharge, the physician assigns an ICD-9 code for the diagnosis and a colour code that indicates the clinical severity of the patient.

The fast-track process that relates to specialist consultation is applicable only during the working time of specialists (between 8 am and 8 pm from Monday to Friday, and from 8 am to 2 pm on Saturday). However, the fast track process related to minor trauma that requires an X-ray examination is applicable 24/24 h due to the presence of a Radiology service within the Emergency Department.

The rules for the fast-track process are defined in regional law published in the local regional bulletin [13]. The installation of the fast track process was resource neutral and no more specialists were hired to allow the activation of the fast-track process.

### Measurements

Demographic characteristics (age, gender) were considered.

When the patient arrives to the ED, the colour code is assigned by a skilled nurse during the triage evaluation following the current Italian guidelines. The colour code sets the priority of the ED physician’s visit. There are four levels of priority: white tag (non-urgent condition); green tag (less urgent condition/low priority); yellow tag (urgent, potentially life-threatening emergency condition); or red tag (very critical, immediately life-threatening emergency condition). The 4 level Italian system had demonstrated a good inter-rater and intra-rater reliability rating triage acuity and accuracy in patient admission and prediction [14].

The fast-track process can be activated only for patients admitted with a low priority code. For this reason, we considered only patients admitted with white or green code. The final diagnosis (made by the ED physician at discharge) Has been formulated in accordance with the ICD-9 classification [15].

### Outcomes

To evaluate the efficiency of the fast-track process we considered the waiting time before the clinical examination by the ED physician, the time between clinical examination and discharge, and the total length of stay in the ED. The electronic ED system provides the exact time when the patient is admitted, when the clinical examination starts, and when the patient is discharged. The safety of the fast-track process was evaluated as the number of unplanned readmissions after discharge from the ED at three time points, i.e. within 48 h, 7 and 30 days.

### Sample size

Previous studies found a decreased length of stay in the ED of 15% in the fast-track process group [4]. This parameter was used to calculate the sample size. The mean time spent in the ED by older adults treated and released from 2010 to 2012 was 209 min ±127 SD. In view of a difference between the FT group and the control group of 31 min, we needed to study 264 fast-track subjects and 264 controls, considering an alpha error of 0.05 and beta error of 0.2 with a power of 80%.

Since we used a non-parametric test to compare the length of stay (due to the unusual distribution), we added an extra 10% of subjects [16]. Therefore, each group included 289 subjects. Our matching found 504 subjects in each group, thus exceeding the minimum number that needs to be considered. The sample size estimation was calculated using Power and Sampling size Program version 3.1.6 for Windows®.

The sample was built according to a case-control 1:1 design. Within each year, subjects were stratified based on 4 variables (sex, age, triage category and ICD-9 discharge diagnosis), so we obtained 819 strata for 2010 (controls) and 162 strata for 2012 (cases). The introduction of visiting hours, through a further covariate, resulted in an increase in the number of strata without making any substantial changes to the final date set. Later, we randomly extracted without repetition one control for each case. The final data set counts 141 strata and 1008 observations: 504 cases and 504 controls.

### Analysis

Mean and standard deviation or number and percentages were used to describe the characteristics of the sample, as appropriate. Median and interquartile ranges are used for not-normally distributed variables. The distribution of continuous variables was assessed using the Kolmogorov Smirnov test. Continuous variables with normal distribution were compared using the Student T test for independent sample (fast-track group and controls). Variables not-normally distributed (LOS, waiting time before clinical examination, time between clinical examination and discharge) were compared using the Mann Whitney U test for independent samples. Chi square was used for categorical variables and Fisher’s test was chosen when the expected frequencies were less than 5. Statistical significance was defined as a p level < 0.05. The analysis was performed using SPSS version 24 (Statistical Package for the Social Sciences, IBM, Chicago, Illinois).

## Results

The characteristics of the two groups are presented in Table 1.

**Table 1:** Characteristics of the sample

	Usual process ***N*** = 504	Fast-track process ***N*** = 504	P
**Age**	**74.9 ± 6.92**	**74.9 ± 6.92**	**n.s.**
**Gender (F)**	**315 (62.5) 313**	**315 (62.5)**	**n.s.**
**Colour tag at admission**			**n.s.**
**White**	**19 (3.8)**	**19 (3.8)**	
**Green**	**485 (96.2)**	**485 (96.2)**	
**Diagnoses**			**n.s.**
injuries, poisoning and violence	**376 (74.6)**	**376 (74.6)**	
diseases of the nervous system and sense organs	**86 (17.1)**	**86 (17.1)**	
diseases of the musculoskeletal system and connective tissue	**21 (4.2)**	**21 (4.2)**	
symptoms, signs, and ill-defined conditions	**8 (1.5)**	**8 (1.5)**	
diseases of the genitourinary system	**4 (0.8)**	**4 (0.8)**	
diseases of the digestive system	**4 (0.8)**	**4 (0.8)**	
neoplasms	**2 (0.4)**	**2 (0.4)**	
infectious and parasitic diseases	**1 (0.2)**	**1 (0.2)**	
diseases of the circulatory system	**1 (0.2)**	**1 (0.2)**	
diseases of the blood and blood-forming organs	**1 (0.2)**	**1 (0.2)**	
**FAST-TRACK process**			
**X-ray examination**	**381 (75.6)**	**–**	
**Specialist consultation**	**123 (24.4)**	**–**	
Ophthalmologist	93 (75.6)	**–**	**–**
Ear-nose -throat	23 (18.7)	**–**	
Gynaecologist	7 (5.7)	**–**	

The mean age was 75 years and there was a predominance of women. Most of the subjects were classified as less-urgent (green) tag.

Table 2 shows differences in time spent in ED: total length of stay (time between admission and discharge), the time spent between admission and ED physician clinical examination, and between ED physician clinical examination and discharge.

**Table 2:** Time spent in the Emergency Department in the fast-track group and in control group

	Usual process ***N*** = 504	Fast-track process ***N*** = 504	P
**(median, IQ range)**			
**ED Length of stay (minutes)**	**178 (184) 264**	**115 (99)**	**< 0.001**
**Time between admission and ED physician clinical examination (minutes)**	**83 (134)**	**100 (83)**	**< 0.001**
**Time between ED physician clinical examination and discharge (minutes)**	**78 (120)**	**3 (6)**	**< 0.001**

The LOS is significantly lower in the fast-track group, as well as the time spent between the ED physician’s visit and the discharge. Also of note, is the longer time between admission and the ED physician’s visit being recorded, but this time was spent doing an X-ray exam or a specialist consultation.

The rate of the readmission is low in both groups (Table 3)**.** Despite this, a significant difference was seen between the two groups at seven and thirty days. The rate of the readmission in the fast-track group is lower than 1% within the three time points, staying below 1% within 30 days, compared with a rate of readmission of 6.5% in the control group (*p* < 0.001).

**Table 3:** Readmission rate at different time points in patients in the fast- track group and in the control group

	Usual process ***N*** = 504	Fast-track process ***N*** = 504	P
**Readmission within 48 h (n, %)**	**0 (0)**	**0 (0)**	**n.s.**
**Readmission within 7 days (n, %)**	**5 (0.9)**	**0 (0)**	**< 0.001**
**Readmission within 30 days (n, %)**	**34 (6.5)**	**5 (0.9)**	**< 0.001**

## Discussion

Our results show that patients who are managed through the fast-track process had a reduction of 36% in the ED length of stay. The number of unplanned readmissions was low in both groups, without any increase in the fast-track group. These findings support the efficiency and safety of the fast-track process in older patients. This is extremely important considering the detrimental effects of long ED LOS in these patients.

Driesen et al. found that patients who exceeded an ED-LOS > 6 h were generally more complex and older, but many root causes contributing to an increased ED-LOS were related to organizational factors, including radiological imaging or sequential specialist consultations [17].

Long completion time in the ED is associated with negative outcomes during the ED stay, such as increased risk of hospital admission and in-hospital mortality [6, 7], with a significant decrease in self-rated health, due to the loss of functional capacity [18]. Many reviews identify the fast-track process as an effective approach to reduce the length of stay in the ED, as well as ED overcrowding, without affecting the quality of care [2, 19–22]. Although there is an extensive body of literature, to the best of our knowledge, no study focused on older adults.

The reduction in the length of stay of patients managed via fast-track varies in different studies, ranging from 20 to more than 60 min [3, 4]. In our sample we found a significant decrease in the time spent in the ED, compared with studies which considering the general population [4].

Moreover, the fast-track process seems safe due to the readmission rates even lower than in the usual care group.

Our study has some limitations that should be acknowledged. First, it is monocentric, and it has a retrospective design. For these reasons, our results should be considered as preliminary. Also, our methodology to detect readmissions, based on the hospital database, ignores the possibility that patients are readmitted to other hospitals. However, the hospital in Senigallia serves an area of about 45,000 subjects, and older patients living in the area are usually admitted to it. The nearest hospital is about 30 km distant. This makes it less likely that older patients were referred to other facilities.

The literature describes different models of the fast-track system. In our ED there isn’t a specific area to manage the fast-track patients, and the final discharge is decided by the ED physician. Moreover, our data are quite old, because we decided to compare data gathered during the year before and after the introduction of the FT process, to avoid possible bias of selection using data collected during the same year. Finally, the rate of readmission is low and deserves caution in the interpretation.

## Conclusions

In summary, the fast-track appears to be a useful and safe strategy to improve the management of older adults admitted to the ED with minor complaints. Further studies are essential to confirm these results, considering the increasing need of strategies to reduce the overcrowding and improve the quality of care for older adults in the emergency department.

## Data Availability

The data that support the findings of this study are available from Direction of the Hospital Principe di Piemonte but restrictions apply to the availability of these data, which were used under permission for the current study, and so are not publicly available. Data are however available from the authors upon reasonable request and with permission of Direction of the Hospital Principe di Piemonte.

## Abbreviations

FT: Fast track
LOS: Length of stay
ED: Emergency Department
